# A 10-Year Surveillance of *Eimeria* spp. in Cattle and Buffaloes in a Mediterranean Area

**DOI:** 10.3389/fvets.2020.00410

**Published:** 2020-08-04

**Authors:** Maria Elena Morgoglione, Antonio Bosco, Maria Paola Maurelli, Leucio Camara Alves, Giorgio Saralli, Gianpaolo Bruni, Giuseppe Cringoli, Laura Rinaldi

**Affiliations:** ^1^Department of Veterinary Medicine and Animal Production, University of Naples Federico II, CREMOPAR Campania Region, Naples, Italy; ^2^Department of Veterinary Medicine, Federal Rural University of Pernambuco, Recife, Brazil; ^3^Istituto Zooprofilattico Sperimentale del Lazio e della Toscana M. Aleandri, Rome, Italy

**Keywords:** eimeriosis, seasonal dynamics, cattle, buffaloes, Mediterranean area

## Abstract

Coccidiosis due to *Eimeria* spp. are widespread parasitic infections in cattle and water buffaloes and may impair health, welfare, and production of these livestock species. The aims of this study were (i) to investigate the prevalence and seasonal dynamics of eimeriosis and (ii) to characterize the *Eimeria* species in large ruminants in a Mediterranean area, in order to plan effective control strategies. Parasitological data were obtained from a 10-year surveillance program (2010–2019) on 3,631 farms (2,089 buffalo and 1,542 cattle farms) sampled in central and southern Italy. Pooled fecal samples were analyzed using the FLOTAC technique with an analytic sensitivity of 2 oocysts per gram of feces (OPG) utilizing a saturated sodium chloride flotation solution (specific gravity = 1.200). *Eimeria* species identification was performed by morphometric analysis after a one week incubation of oocysts in a 2.5% potassium dichromate solution. The results showed high prevalence of *Eimeria* (up to 100%) in both cattle and buffaloes in the 10 years of surveillance, even if a slight reduction was reported in the last three years. The overall prevalence of eimeriosis was 91.7% (95% confidence interval, 95% CI = 90.2–93.1) in cattle farms and 81.5% (95% CI = 79.8–83.1) in water buffalo farms. The mean OPG value was 66.8 (min = 2; max = 8,065) in cattle and 55.9 (min = 2; max = 15,415) in water buffaloes, but this difference was not statistically significant (*p* > 0.05). In total, nine species of *Eimeria* were found in cattle the most prevalent being *Eimeria bovis, E. ellipsoidalis, E. cylindrica*, and *E. zuernii*, whereas in water buffaloes eight species of *Eimeria* were found, the most prevalent being *E. ellipsoidalis, E. auburnensis, E. bovis*, and *E. zuernii*. Mixed infections were common in both ruminant species. The seasonal pattern showed a higher prevalence of eimeriosis in cattle in spring (86.9%) whereas in buffalo farms the prevalence was higher in winter (82.3%) and summer (82.4%). In conclusion, the 10-year surveillance program indicates that eimeriosis is common in cattle and water buffaloes and therefore continuous effective control strategies are needed.

## Introduction

Coccidiosis due to *Eimeria* spp. are widespread parasitic infections in cattle and water buffaloes and may impair health, welfare, and production of these livestock species (1–3). Animals become infected by the horizontal route, ingesting sporulated oocysts from contaminated feed, water, or pasture or by licking contaminated hair coat (1, 3, 4). Outbreaks in cattle and water buffaloes are associated with several factors, including the species of *Eimeria*, the age of the animals, immunological status of hosts, the dose of the oocysts ingested, and farm management and environmental factors (5–7).

More than 20 *Eimeria* species are described in cattle (8), and among them, 12 species can affect also water buffaloes (*Bubalus bubalis*) (9, 10) although coccidia are usually host-specific parasites. *E. zuernii, E. bovis*, and *E. auburnensis* are the most pathogenic species in both hosts worldwide (11, 12), while *E. bareillyi* is a pathogenic species specific only for water buffaloes (13).

Usually adult animals are asymptomatic, although they can be a reservoir for younger ones (14, 15), whereas calves can show gastrointestinal (GI) signs, such as diarrhea, dysentery, dehydration, debilitation, and even death (5, 8).

Compared with cattle, there is limited scientific knowledge about the health of water buffaloes so updated data on parasitic infections (as eimeriosis) is an interesting challenge in this species where knowledge regarding the health consequences of the most common pathologies as well as their economic impact on the entire dairy food chain are still almost rare (16).

Indeed, considering the health and welfare implications, as well as the economic losses due to *Eimeria* infections in ruminant livestock, the knowledge of their geographical distribution, prevalence, and intensity of infection is important to understand the dynamic of infection in relation to biotic (such as age) and abiotic (such as seasonality) factors (6) especially in areas where dairy cattle and water buffalo farms coexist and play a major role for the economy of the region (16). The published studies on eimeriosis in large ruminants in Italy are few and focused mainly on treatment (17–19), while the epidemiological data in Europe are scarce, not updated, and focused only on cattle (11, 20–23).

For these reasons, the aims of this study were (i) to investigate the prevalence and seasonal dynamics of eimeriosis and (ii) to speciate the *Eimeria* in large ruminants in a Mediterranean area, in order to plan effective control strategies.

## Materials and Methods

### Study Area and Design

The study was conducted in three Italian regions: Lazio (latitude = 41°53′35″N; longitude = 12°28′58″E) in the Center, Campania (latitude = 40°49′34″N; longitude = 14°15′23″E) and Basilicata (latitude = 40°38′21″N; longitude = 15°48′19″E) in the South. The study area extends over 40,898 km^2^ from the Apennines to the Tyrrhenian Sea where cattle and water buffaloes are bred. The entire area is characterized by high heterogeneity with hills and mountains inland and lowlands mainly near the coast. This area is characterized by mild and wet autumns/winters with an average monthly temperature of 9°C and hot and dry springs/summers with an average monthly temperature of 22°C (24).

**Figure 1:**
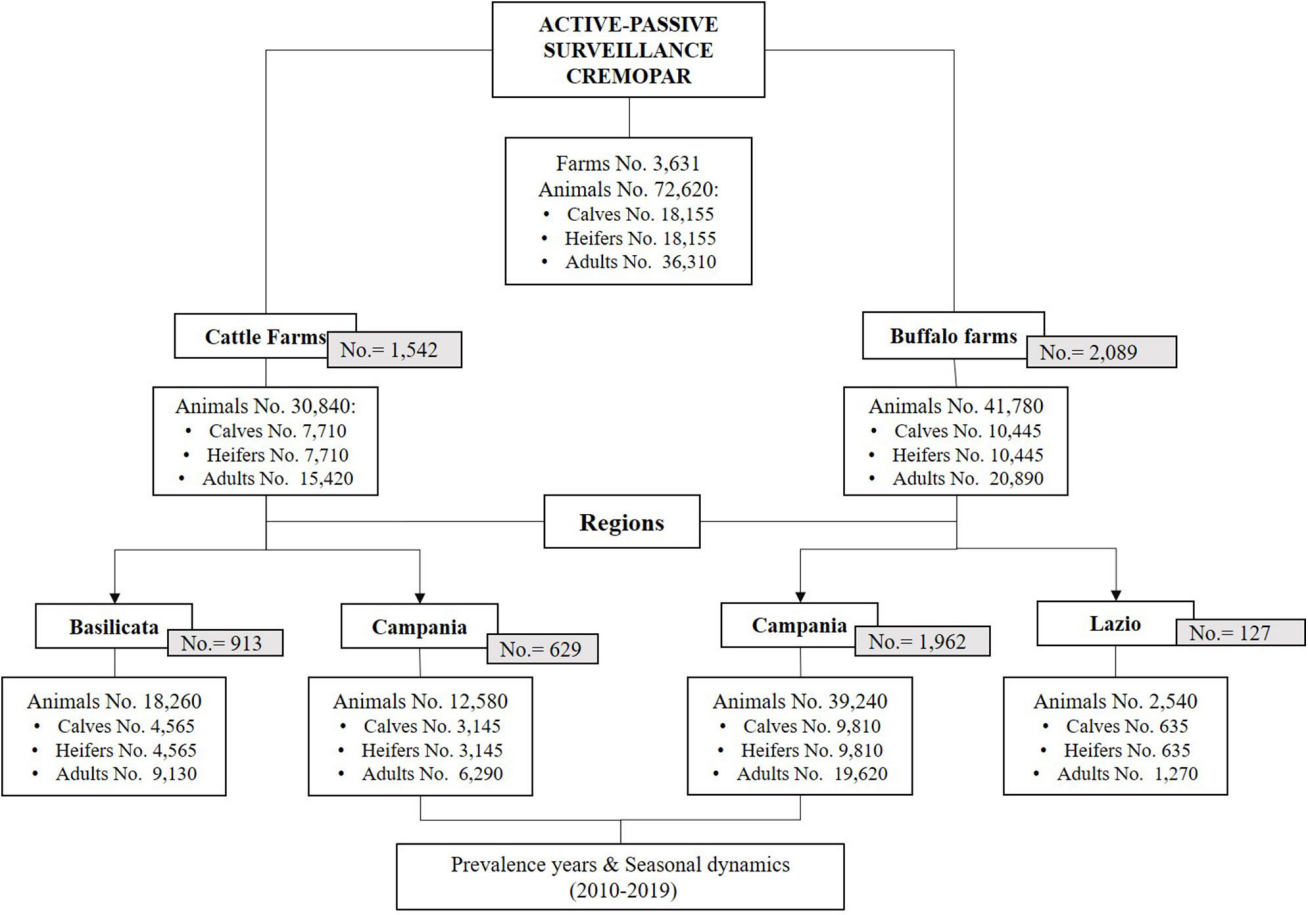
Study setup of the analyzed data from the 10-year surveillance program, with the total number of cattle and water buffalo farms, total number of animals, and age categories in the Italian regions involved (Lazio, Campania, and Basilicata).

Parasitological data were obtained by the Regional Centre for Monitoring of Parasitosis (CREMOPAR, Campania Region, Southern Italy) from a 10-year program (2010–2019) of active and passive surveillance on 3,631 farms (cattle and water buffalo farms) (Figure 1). Data related to cattle farms in the Lazio region and water buffalo farms in the Basilicata region were fragmented, so they were not included in the study. Moreover, analysis of yearly prevalence and seasonal dynamics of cattle and buffalo coccidiosis was performed only in Campania region, because full data were available through all the years of this study, due to the continuous monitoring service offered by the Department of Agriculture of the Campania Region, through the activities of CREMOPAR.

### Farm Management

#### Cattle Farms

Cattle (*Bos indicus* and *B. taurus*) are the most common world widespread species of large ruminant livestock. Cattle are raised in diverse production systems ranging from capital-intensive, specialized beef and dairy grass-based and feedlot systems (25).

**Figure 2:**
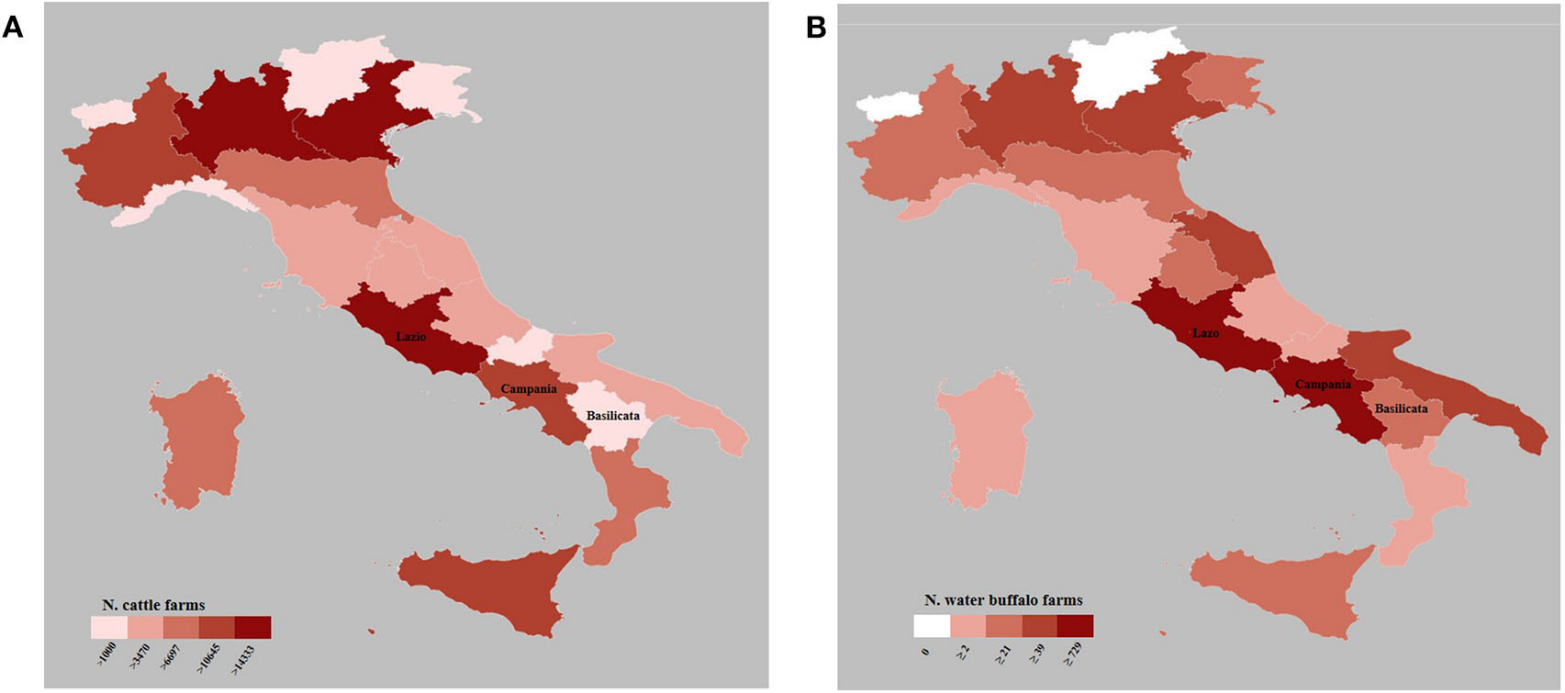
Maps of distribution of cattle **(A)** and buffalo **(B)** farms in Italy at 31th December 2019 (data by National Data Bank).

In the study area, cattle are raised for meat and/or milk production. The dairy farms are characterized by an intensive farming system, with suitable buildings and modern equipment to guarantee animal welfare, in order to maximize the production (26). On the other hand, the meat production is mainly characterized by an extensive farming system, with daily grazing and sheltering in part-time housing. This system allows the animals to graze on poor soils with minimal vegetation. In the study area, the two productive realities coexist: dairy farms are spread in the plain and in the foothills area, while the beef cattle are on grazing and marginal land. The Italian cattle population amounts to more than 5 million onto 145,363 farms (Figure 2A). The numbers of cattle farms in Campania and Basilicata represent 7.3% and 1.9% of the Italian farms, respectively (National Data Bank—NDB at 31th December 2019).

#### Buffalo Farms

Water buffalo (*B. bubalis*) farming is important for the economy of several countries, including Brazil, China, India, Vietnam, and Italy. Mozzarella cheese manufacturing from milk of water buffalo is third-ranked in sales volume in Italy (27). The modern intensive water buffalo breeding is likely to replace the cattle breeds and has almost completely replaced the traditional free-range/semi-free-range buffalo farming (21, 28). Currently, the buffalo management is characterized by technologically advanced and automatic systems (e.g., milking robots, automatic manure cleaning, the use of the pedometer for individual measurements of physiological/production parameters, etc.). The southern provinces of Lazio (Latina and Frosinone), the Campania region, and other two southern provinces not included in the study area (Foggia and Isernia) represent the area of buffalo mozzarella cheese with the Protected Designation of Origin (PDO) mark (29). In Italy, there are 2,711 buffalo farms (Figure 2B) with a total of 402,796 animals. Lazio and Campania are the regions with the highest percentage of the total buffalo farms in Italy with 26.9% and 48.8%, respectively (NDB at 31th December 2019).

### Copromicroscopic Analysis

A total of 72,620 fecal samples were collected directly from the rectum of animals involved in the study. In each farm, individual fecal samples (at least 20 g) from 20 animals were collected according to three age groups: 5 calves (0–6 months), 5 heifers (7–12 months) and 10 adults (>12 months). The collected samples were stored by *vacuum* packaging (30) and sent to the laboratories of CREMOPAR. In the laboratory for each farm, 4 pools of feces (one for calves, one for heifers, and two for adults) were prepared, taking 5 g of each individual fecal sample (31). Pooled samples were analyzed by the FLOTAC technique with an analytic sensitivity of 2 OPG, using a sodium chloride flotation solution (specific gravity = 1,200) (32).

In order to sporulate the oocysts and identify the *Eimeria* species, the fecal samples from each positive farm (OPG ≥50) were pooled into one sample (at least 10 g), diluted 1:10 with a 2.5% potassium dichromate solution and incubated in a container at 26–28°C for one week, oxygenating the samples several times a day (33). The *Eimeria* species were identified using the morphometric keys of Eckert et al. (34) and de Noronha et al. (33).

### Statistical Analysis

Chi-square (χ^2^-test) was employed to verify the association between prevalence and age group of animals and between prevalence of different *Eimeria* species and regions for both hosts. One-way ANOVA test was performed to detect OPG variability between seasons through the years. Difference was considered significant at *P* < 0.05. These statistical analyses were performed with SPSS 23.0 software (IBM, Armonk, NY, USA).

## Results

### Prevalence of Eimeriosis

**Figure 3:**
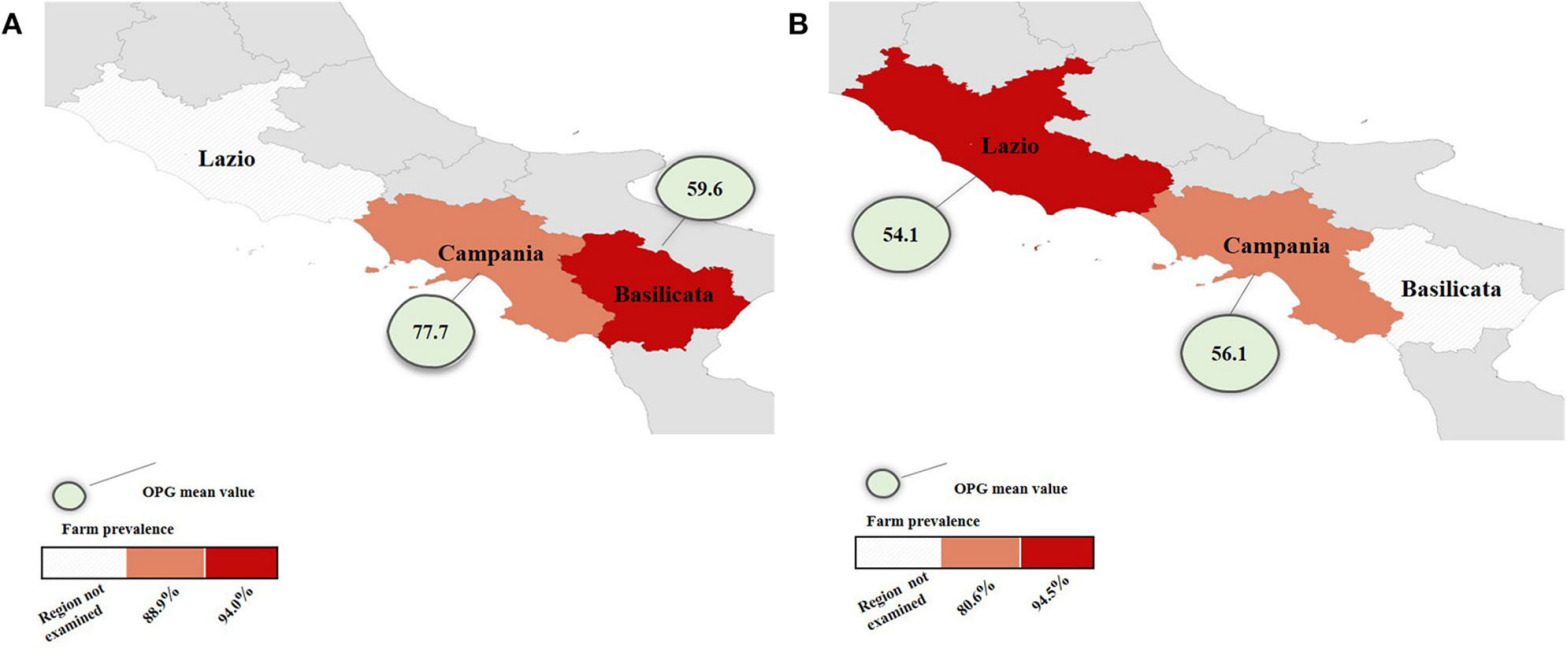
Maps of farm prevalence and OPG mean value of cattle **(A)** in Basilicata and Campania and water buffalo **(B)** farms in Lazio and Campania.

*Eimeria* spp. was found in both cattle and water buffaloes showing a prevalence of 91.7% (95% confidence interval, 95% CI = 90.2–93.1) in cattle farms and 81.5% (95% CI = 79.8–83.1) in water buffalo farms with statistically significant difference (*P* < 0.05). In buffaloes from Lazio, the prevalence was higher than in the Campania region with a statistically significant difference (*P* < 0.05). Regarding OPG, the overall mean value was 66.8 in cattle and 55.9 in water buffaloes, but this difference was not statistically significant (*P* > 0.05). These results were represented in Figures 3A,B. The highest prevalence rate and OPG mean values were recorded in young animals (Table 1). The one-way ANOVA *test* showed that calves had OPG values significantly higher (*P* < 0.05) in both cattle and buffalo farms.

**Table 1:** Farm prevalence (95% CI) of *Eimeria* spp., age-group mean OPG, minimum, and maximum OPG values, in cattle farms in Campania and Basilicata regions and in buffalo farms in Lazio and Campania regions.

**Region**	**Host**	**Farm prevalence% (95% CI)**	**Oocysts per gram of feces (farm prevalence%)**				
			**Mean (prevalence%)**			**Minimum**	**Maximum**
			**Calves**	**Heifers**	**Adults**		
Campania	Cattle	88.9 (86.1–97.1)	167.2 (61.0)	65.6 (67.8)	40.1 (38.8)	2	8,065
Basilicata	Cattle	94.0 (92.2–95.4)	188.3 (89.9)	47.4 (87.7)	45.6 (39.8)	2	8,005
Lazio	Water buffalo	94.5 (88.6–97.6)	145.0 (83.3)	46.8 (83.3)	17.0 (74.2)	2	2,250
Campania	Water buffalo	80.6 (78.9–82.3)	256.1 (80.3)	39.7 (77.7)	24.8 (51.8)	2	15,415

### Yearly Prevalence and Seasonal Dynamics of Infection

Yearly prevalence of positive farms to *Eimeria* infection showed a mean of 86.4% in cattle farms and 82.1% in water buffalo farms. A higher coccidiosis prevalence (100%) was reported in cattle farms from 2012 to 2013, in water buffalo farms from 2012 to 2014. Despite the high prevalence of eimeriosis registered every year, a trend of decrease was recorded in the last three years (from 2017 to 2019) in both hosts.

The general pattern of the excreted mean OPG was very irregular in both hosts. From 2010 to 2013, the values recorded in cattle and water buffaloes were similar, while the highest OPG values were reported in cattle in 2017 and in water buffaloes in 2016.

Although the annual mean prevalence was highest in spring (86.9%) in cattle farms while in water buffalo farms was highest in summer (82.4%) and winter (82.3%), no statistically significant differences (*P* > 0.05) between seasons were found in either hosts.

### Identification of *Eimeria* Species

Nine species of *Eimeria* were found in cattle and eight in water buffaloes (Table 2). *E. bovis* and *E. zuernii*, the most pathogenic species in cattle, were present in both hosts and in all the three studied regions. *E. bareillyi*, host-specific and pathogenic for buffalo, was found in Lazio and Campania regions with a prevalence of 13.0 and 11.0%, respectively. Mixed infections were common in both livestock species; in particular, 71.2% of cattle and 39.4% of water buffalo farms were infected with more than one *Eimeria* species. In cattle, the prevalence of *E. subspherica, E. zuernii, E. bovis, E. canadensis*, and *E. alabamensis* was higher (*P* < 0.05) in Basilicata than in the Campania region.

**Table 2:** Prevalence of *Eimeria* species identified in cattle farms in Campania and Basilicata regions and in water buffalo farms in Lazio and Campania regions.

**Region**	**Basilicata**	**Campania**		**Lazio**
**Host**	**Cattle**	**Cattle**	**Buffalo**	**Buffalo**
	**Prevalence% (95% CI)**			
***Eimeria*** **species**				
E. subspherica^*^	26.2 (20.2 – 33.1)	12.5 (7.1 – 20.8)	18.7 (13.1 – 25.9)	17.2 (10.6–26.4)
E. zuernii^*^	40.3 (33.4 – 47.7)	20.2 (13.2 – 29.4)	18.1 (12.5 – 25 – 2)	18.2 (11.4–27.5)
E. ellipsoidalis^*^	43.5 (36.4 – 50.8)	34.6 (25.7 – 44.7)	36.1 (28.7 – 44.3)	36.4 (27.1–46.7)
E. cylindrica^*^	36.1 (29.4 – 43.4)	26.9 (18.9 – 36.7)	0.0	0.0
E. alabamensis^*^	6.3 (3.4 – 11.0)	1.0 (0.1 – 6.0)	0.0	0.0
E. bovis^*^	78.0 (71.3 – 83.5)	57.7 (47.6 – 67.2)	21.3 (15.3 – 28.7)	23.2 (15.6 – 3 3.0)
E. canadensis^*^	12.0 (7.9 – 17.7)	2.9 (0.8 – 8.8)	0.0	0.0
E. wyomingensis^*^	7.9 (4.6 – 12.9)	7.7 (3.6 – 15.0)	9.7 (5.7 – 15.7)	0.0
E. auburnensis^*^	8.9 (5.4 – 14.1)	10.6 (5.6 – 18.5)	27.1 (20.4 – 34.9)	26.3 (18.2–36.2)
E. brasiliensis^*^	0.0	0.0	0.0	0.0
*E. pellita*	0.0	0.0	3.2 (1.2 – 7.8)	0.0
E. bukidnonensis^*^	0.0	0.0	0.0	0.0
*E. bareillyi*	–	–	11.6 (7.2 – 18.0)	13.0 (10.8 – 15.9)

* *Eimeria species common to cattle and buffalo*.

## Discussion

The 10-year surveillance program indicates that eimeriosis is common (up to 100%) in cattle and water buffaloes in the Mediterranean area studied as in different parts of the world (5, 7, 8, 11, 13, 15, 35, 36). The overall prevalence of *Eimeria* spp. was higher in cattle farms (91.7%) than in water buffalo farms (81.5%). These findings could be explained by the best management practices of modern intensive water buffalo breeding. In particular, the mean coccidiosis prevalence in cattle farms reported in the Campania region in our study (88.3%) was lower than the value of 100% detected in a previous study performed in extensive farms in southern Italy (20). For water buffalo farms, the mean prevalence (80.6%), in the decade 2010–2019, showed a small reduction compared to 97.7% reported in the previous decade (2000–2009) in the Campania region (21, 37). Therefore, these results are in agreement with the earlier findings of the 10-year analysis, showing that the epidemiology of *Eimeria* spp. in this study area has changed over time with a slight reduction in the last three years. This decrease may be due to a control plan implemented by CREMOPAR which started in 2014 through the Rural Development Programme (38) of Campania Region aimed to promote regular and accurate parasitological diagnosis, treatment strategy, and dissemination of best practices of management to cattle and water buffalo farmers. Nonetheless, *Eimeria* is still widespread in the cattle and water buffalo farms.

The mean OPG value was 66.8 (min = 2; max = 8,065) in cattle and 55.9 (min = 2; max = 15,415) in water buffaloes, but this difference was not statistically significant (*P* > 0.05). The mean OPG levels were statistically higher in calves (174.3) than in adult animals (43.2), in both livestock hosts, in agreement with other studies performed in cattle in different countries as Pakistan (39), Germany (40), Kenya (5), and Mexico (7) and in water buffaloes in Brazil (33) and in Pakistan (41). The results of seasonality showed there were no significant differences between the seasons.

Some authors found statistically significant differences between seasons and prevalence in animals (1, 7, 8, 35, 41, 42), but in the Mediterranean area the large ruminant farming system is mainly intensive and so the presence of *Eimeria* might not be influenced by the weather or by grazing, but rather by overcrowding and herd management (e.g., hygiene of pens).

The most prevalent species of *Eimeria* found in this study were *E. bovis* (67.9%), *E. ellipsoidalis* (39.1%), *E. cylindrica* (31.8%), and *E. zuernii* (30.3%) in cattle. These species were widespread also in other countries (8, 12, 36, 43, 44), while some species, such as *E. pellita, E. bukidonensis*, and *E. brasiliensis* (7, 8, 12, 43, 44), were not found in our study. In water buffaloes, *E. ellipsoidalis* was the most prevalent (36.3%) species, followed by *E. auburnensis* (26.7%), *E. bovis* (22.3%), and *E. zuernii* (18.2%); in addition, *E. bareillyi*, the buffalo host-specific species, showed a prevalence of 12.3%. These *Eimeria* species were found also in other countries, such as Netherlands, Egypt, Turkey, Iran, Pakistan, India, and Brazil (13, 35, 45), while *E. cylindrica, E. alabamensis, E. canadensis, E. brasiliensis*, and *E. bukidnonensis* found by several authors (13, 35, 45) were not found in our study. Mixed infections with more than one species were common in cattle and buffalo farms with values of 71.2 and 39.4%, respectively. Of the *Eimeria* species detected in this study, only *E. bovis, E. zuernii, E. auburnensis*, and *E. bareillyi* are responsible of severe clinical disease due to intestinal lesions with effects on the digestive process and overall homeostasis (46). However, the presence of clinical eimeriosis was not assessed in this study and further research is needed to investigate the effects of different species and OPG level on disease development in cattle and buffaloes.

*Eimeria* species in cattle and water buffalo are identified only through morphological characteristics, but to date there are no studies showing that species in cattle are genetically identical to the ones in water buffaloes. For this reason, molecular techniques using the 18S ribosomal RNA (rRNA) region can be used, not only to identify *Eimeria* species but also to study intra- and inter-genetic variations in cattle and water buffalo species (47, 48). The accurate identification of *Eimeria* species has important implications for disease control (49), selection of treatment strategies [e.g., metaphylactic treatments; (50)], and identification of alternative therapeutic approaches [e.g., ozone and intestinal microbiome; (51, 52)].

Metaphylactic treatments with toltrazuril was very useful against *Eimeria* infections in cattle (18, 50, 53), as well as in water buffaloes (19), showing improved performances in animals (e.g., faster body weight gain, positive influence on the average age at the first birth, increased overall percentage of pregnancies). Moreover, a reduction in oocyst excretion was demonstrated, with particular reference to the two species considered to be mainly responsible for clinical coccidiosis (*E. zuernii* and *E. bovis*) (18). Therefore, the metaphylactic approach should also contribute to the reduction in environmental contamination with oocysts, limiting the infection pressure (18, 54). However, the efficacy of toltrazuril could be increasingly reduced by the development of *Eimeria* resistance in ruminants (55). Thus, new low-cost and eco-friendly anti-*Eimeria* strategies are urgently required. Alternative therapeutic approaches based on ozone in ruminants (51) could be useful to control *Eimeria* infections as demonstrated in poultry (56). Moreover, recent studies have highlighted the complex network of interactions occurring between protozoa and the gut commensal flora, showing the potential contribution of the intestinal microbiome in the control of parasitic infections (52).

In conclusion, the findings obtained showed that the coccidiosis is a persistent and complex problem, so a combination of good management practice, affordable diagnostic techniques, and strategic treatments (traditional and/or alternative) could be useful to plan an effective control of *Eimeria* infections in large ruminants.

## Data Availability Statement

The raw data supporting the conclusions of this article will be made available by the authors, without undue reservation.

## Ethics Statement

This animal study was reviewed and approved by Ethic Committee of the Department of Veterinary Medicine and Animal Production, University of Napoli Federico II. Written informed consent was obtained from the owners for the participation of their animals in this study.

## Author Contributions

LA, GS, GB, GC, and LR contribute to the conception and design of the study. MEM and AB organized the database. MEM, AB, MPM, and LR wrote the manuscript. MEM and MPM performed the statistical analysis and GIS. All authors contributed to manuscript revision and read and approved the submitted version.

## Conflict of Interest

The authors declare that the research was conducted in the absence of any commercial or financial relationships that could be construed as a potential conflict of interest.
